# Understanding the Emotional Labor of Public Health Equity Work: a Mixed Methods Study

**DOI:** 10.1007/s40615-022-01292-9

**Published:** 2022-03-31

**Authors:** Chad Abresch, Carol Gilbert, Marilyn Johnson, Bibhusha Karki, Kiara Lyons, Karly Meyer, Melissa Tibbits, Drissa Toure

**Affiliations:** 1 Department of Pediatrics, College of Medicine, University of Nebraska Medical Center, Omaha, NE USA; 2 Department of Health Promotion, College of Public Health, University of Nebraska Medical Center, Omaha, NE USA; 3 Department of Epidemiology, College of Public Health, University of Nebraska Medical Center, Omaha, NE USA; 4 College of Medicine, University of Nebraska Medical Center, Omaha, NE USA; 5 Department of Education and Child Development, Munroe-Meyer Institute, University of Nebraska Medical Center, Omaha, NE USA

**Keywords:** Public health, Equity, Emotional labor, Workforce development, Burnout, Job satisfaction

## Abstract

**Background:**

Rectifying historic race-based health inequities depends on a resilient public health workforce to implement change and dismantle systemic racism in varied organizations and community contexts. Yet, public health equity workers may be vulnerable to job burnout because personal investment in the continual struggle against inequality exacts an emotional toll. Our study sought to quantify the presence of emotional labor in public health equity work and better understand its dimensions.

**Methods:**

We conducted a mixed methods study of public health equity workers focused on maternal and child health in the USA. Participants completed a survey on the emotional demands of their public health equity work. A subset of survey respondents was interviewed to gain a better understanding of the emotional toll and support received to cope.

**Results:**

Public health equity work was found to involve high levels of emotional labor (*M* = 5.61, range = 1–7). A positive association was noted between personal efficacy (i.e., belief in one’s ability to do equity work well) and increased job satisfaction. However, burnout increased when equity workers did not receive adequate support for their emotional labor. Qualitative analysis revealed eight themes depicting the emotional burden, benefits and drawbacks, and coping strategies of public health equity work.

**Conclusions:**

Public health equity workers report high degrees of emotional labor and inadequate workplace support to cope with the demands. In our study, workplace support was associated with higher job satisfaction and lower burnout. Research is urgently needed to develop and scale an effective model to support public health equity workers.

## Background

As a profession, public health has long been recognized as work that requires personal investment [1]. The pandemic and racial justice movements of the past 18 months have served only to heighten the personal burden experienced by the public health workforce [2]. Recent reports suggest that burnout in the field has increased to a level causing significant strain on the public health sector [3, 4].

For those working specifically in the sphere of health equity, the personal burden is likely to be intensified. As Braveman [4p371] explains, “It should be more widely understood that the concepts of health equity and health disparities are not value-neutral, but specifically address social justice in the realm of health.” Braveman goes on to note that health equity is inextricably linked to civil and political rights. Health inequities, therefore, constitute violations of those rights. Consequently, we can understand that public health equity work is the expansive discipline that seeks to correct social injustices so that fair health outcomes can be realized. To be sure, the intense personal investment and emotional burden in such work are significant.

One framework used extensively in the literature for studying personal investment in the workplace is “emotional labor” [5]. Emotional labor occurs when work requires the management of personal emotions for effective job performance or the realization of workplace norms and goals [6–8]. Scholars have found both positive and challenging aspects of emotional labor. For example, Guy and colleagues [9] noted a positive correlation between emotional labor and job satisfaction in a diverse sample of public and nonprofit workers; however, this association is nuanced and inconsistent across the literature [10–12]. Alternatively, scholars have noted negative consequences associated with high levels of emotional labor [13, 14]. Aung and Tewogbola [15] recently conducted a thorough review of the emotional labor literature and found alarming links to adverse health outcomes, including fatigue, loss of sleep, burnout, exhaustion, and depression. For example, one study [16] found nurses’ witnessing of patient suffering resulted in ongoing mental and emotional difficulties accompanied by a sense of being drained of energy. Even more alarming, another study [17] conducted with Korean service and sales workers found high emotional labor demands increased suicidal ideation and, for men, this effect was exacerbated when coupled with low job control.

Given the predominance of these findings, research in several fields, including occupational health and organizational psychology are now beginning to look at factors that may moderate the negative impacts of emotional labor, such as supervisory support, sleep regulation, organizational climate, and emotion regulation [18–20]. Beginning with Grandey [21], a model of emotional labor as emotion regulation within the workplace has been developed, creating new research measures and intervention opportunities.

What has not been studied to this point is emotional labor and its impacts as it relates specifically to public health equity workers. Such research is needed to demonstrate its existence within the field and advocate for workplace programs needed for “maximizing service performance while supporting worker well-being” [26p408].

Our study sought to quantify for the first time the presence of emotional labor in public health equity work and better understand its dimensions. We employed a mixed methods approach to answer the study’s main research question: In what ways is emotional labor required in health equity work, and what does it involve? Additionally, we sought to understand the personal and professional supports public health equity workers need and currently receive.

## Methods

To address these questions, we conducted an explanatory sequential study [22]. The study commenced with a quantitative strand of research that featured a national survey. Three questions guided this portion of our study: (1) how frequently is emotional labor required in health equity work; (2) how does emotional labor impact job satisfaction; and (3) are health equity workers receiving the support they need to cope with the emotional labor involved in their jobs? In the second, qualitative strand of research, interviews were conducted with a subset of survey respondents. Here, questions probed four areas of interest: (1) what does emotional labor involve in public health equity work; (2) what are its rewards and drawbacks; (3) what personal and professional supports are used to cope with the downsides; and (4) do public health equity workers feel enough support is available. The study was reviewed and approved by the research team’s Institutional Review Board (IRB# 628–19-EX).

## Participants

Participants in this study were obtained from purposive and snowball sampling. Specifically, we began by assembling three diverse participant seed lists [23–25], each comprising distinct policy levels within the field of maternal and child health (MCH). We chose to focus on the field of MCH because of the workforce’s frequent involvement in public health equity [26, 27]. The participant seed lists were derived from membership lists of three national MCH organizations, including CityMatCH, which represents local MCH, the Association of Maternal and Child Health Programs, which represents state MCH, and the National Healthy Start Association, which represents community-based MCH. To ensure respondents had a history of direct public health equity work, the three national MCH organizations selected individuals known to be involved in equity initiatives. To conduct the snowball sample, we asked survey respondents to provide three names of colleagues whose work focused on public health equity.

## Data Collection

For the quantitative phase of our study, we employed an online questionnaire via REDCap adapted from a validated instrument: the GNM Emotional Labor Questionnaire [9]. Our Health Equity Emotional Labor Questionnaire consisted of 24 questions in six scale categories. The six scales used in our survey are shown in Table 2 and were consistent with the GNM questionnaire except for the following. In two instances we combined similarly themed scales. Additionally, we added a scale—emotional labor supports—not included in the GNM questionnaire. Questions on our health equity emotional labor questionnaire were also revised slightly to focus directly on public health equity work. Responses were seven-point Likert scales (i.e., always true to never true, strongly agree to strongly disagree, all the time to never). All questions were on a scale of 1 (indicating a negative response) to 7 (indicating a positive response). The final survey used in our study was reviewed by one of the GNM questionnaire developers for adherence to intent and constructs and approved. The survey was fielded between August 2020 and April 2021.

Participants who completed the questionnaire were asked if they would be willing to participate in a follow-up interview to gain a deeper understanding of their experiences with health equity work. Most survey respondents (73%) were willing to be interviewed, allowing us to purposively select an interview cohort with diversity by policy level, gender, race and ethnicity, age, and geography. Interviews were conducted until the investigative team determined the point of saturation had been achieved [28]. This resulted in a total of 19 interviews conducted between October 2020 and June 2021. The interviews followed an interview guide developed by the investigative team with probes for stories and rich detail. All interviews were conducted via Zoom, recorded, and later transcribed for analysis.

## Statistical Analyses

### Phase One-Quantitative Analyses

All analyses for this phase of the study were completed using SAS Studio v 3.8. Descriptive statistics, such as frequencies and rates, were conducted to summarize the categorical variables.

We used Cronbach’s alpha to test the internal consistency of items within our health equity emotional labor questionnaire. A score of at least 0.70 is needed to be considered acceptable [29]. Once internal consistency for each scale was determined, we then calculated the mean scale score for each scale to compare the average response rating. Pearson analysis was done to determine the degree of correlation among the six scales, using a standard *p*-value of 0.05 to determine significance.

### Phase Two-Qualitative Analyses

Four members of the investigative team independently reviewed three selected transcripts and then met to agree upon a final coding scheme. This was done by members of the team presenting themes they had identified within the three transcripts and providing an opportunity for other members to agree, add clarification, and/or dispute themes. Based on this process, a code tree was developed with twelve overarching themes and fourteen subthemes to provide additional perspective and detail. Following this, codes were loaded in MAXQDA, and interviews were divided among the four researchers for analysis.

## Results

In keeping with the nature of the research, our findings are presented sequentially by research strand.

### Quantitative Findings

#### Participants

Participant demographic information is presented in Table 1. A total of 91 participants completed the survey. Most participants were between the ages of 35 and 64 (87%), female (87%), and identified as Black/African American (54%) or White (41%). Half worked in health equity for 10 years or less (50%) and in governmental positions (51%). The size of respondents’ workplaces varied greatly, with a range of < 50 employees (21%) to 5000 + (9%).

**Table 1:** Participant demographics

Age	%(*n*)
25–34	13(12)
35–44	34(31)
45–54	23(21)
55–64	22(20)
65 +	7(6)
Prefer not to answer	1(1)
Gender	
Female	87(79)
Male	12(11)
Transgender	1(1)
Hispanic/Latino	
Yes	10(9)
Race (check all that apply)	
Black/African American	54(49)
White	41(37)
Asian	3(3)
American Indian/Alaskan Native	1(1)
Native Hawaiian/Pacific Islander	1(1)
Other	2(2)
Years working in health equity	
1–5	22(20)
6–10	28(25)
11–15	13(12)
16–20	14(13)
21–25	7(6)
26–30	5(5)
30 +	9(8)
Prefer not to answer	2(2)
Type of position	
Government	51(46)
Nongovernment	49(45)
People in your organization	
< 50	21(19)
50–99	5(5)
100–499	19(17)
500–999	29(26)
1000–4999	15(14)
5000 +	9(8)
Prefer not to answer/missing	2(2)

#### Aspects of Emotional Labor

Table 2 summarizes mean scores for each of the six scales of emotional labor and the individual items that comprise the scales. Overall, the job pride and satisfaction, emotion work, and personal efficacy scales had the highest scores. Participants were less likely to positively endorse burnout, emotional labor supports, and false face scales.

**Table 2:** Aspects of emotional labor

Scales and individual item scores	Mean (range 1–7)	Standard deviation
1. Job pride and satisfaction	5.84	0.85
1a. Health equity work is interesting	6.23	0.78
1b. I am proud of the health equity work I do	6.11	1.01
1c. Health equity is personally satisfying	5.71	0.97
1d. I feel like my health equity work makes a difference	5.47	1.27
2. Emotion work	5.61	0.79
2a. Health equity work produces many different emotions for me	6.15	0.87
2b. Health equity work requires me to guide others through sensitive and/or emotional issues	6.15	0.87
2c. My health equity work involves dealing with emotionally charged issues as a critical dimension of the job	6.00	1.02
2d. Health equity work requires me to deal with unpleasant issues	5.82	1.03
2e. I need to shield myself from feeling the emotions involved in health equity work	3.99	1.64
3. Personal efficacy	5.42	0.83
3a. When appropriate, I am good at helping others realize their role in reducing health inequities	5.75	0.86
3b. When appropriate, I can help others realize how their actions create and sustain health inequities	5.50	1.00
3c. I am good at facilitating difficult conversations related to health equity work	5.46	1.29
3d. I am good at helping others cope with the emotional aspects of health equity work	5.30	1.01
3e. I cope well with the emotional aspects of health equity work	5.08	1.28
4. Burnout	4.13	1.45
4a. Health equity work leaves me feeling emotionally exhausted	4.80	1.55
4b. Health equity work puts a lot of stress on me	4.73	1.74
4c. Health equity work makes me feel used up	4.02	1.78
4d. I worry that health equity work is hardening me emotionally	2.97	1.79
5. Emotional labor supports	4.06	1.14
5a. I receive the emotional support I need from friends and family to perform health equity work	4.47	1.55
5b. I receive the emotional support I need to perform health equity work from other sources other than my employer or friends and family	4.08	1.38
5c. I receive the emotional support I need to perform health equity work	4.03	1.40
5d. I receive the emotional support I need from my employer to perform health equity work	3.67	1.64
6. False face	3.58	1.68
6a. Health equity work requires me to be artificially pleasant when I do not feel that way	3.76	1.72
6b. Health equity work requires that I hide my true feelings	3.41	1.82

#### Relationships Between Emotional Labor Scales

Correlations among the six scales are presented in Table 3. The strongest correlation was between emotion work and burnout (*r* = 0.62, *p* < 0.01). Similarly, the emotion work scale was positively correlated with false face (*r* = 0.41, *p* < 0.01). Personal efficacy was also positively correlated with job pride and satisfaction (*r* = 0.58, *p* < 0.01) and emotional labor supports (*r* = 0.38, *p* < 0.01). Finally, burnout was negatively correlated with emotional labor supports (*r* =  − 0.46, *p* < 0.01).

**Table 3:** Pearson correlation coefficients among the six scales

	Emotion work	Personal efficacy	False face	Burnout	Job pride and satisfaction
Personal efficacy	0.17				
False face	0.41**	− 0.15			
Burnout	0.62**	− 0.07	0.48**		
job pride and satisfaction	0.24*	0.58**	− 0.15	0.07	
Emotional labor supports	− 0.15	0.38**	− 0.37**	− 0.46**	0.28*

^*^p < 0.05; **p < 0.01

### Qualitative Findings

The first strand of research demonstrated that health equity work involves emotional labor and that support for this could be improved. In the second strand of research, we sought to answer additional research questions designed to go beyond the quantitative findings to garner deeper insights from participants.

We identified eight themes related to our research questions, which are discussed below and illustrated with participant quotes (lightly edited for readability). Table 4 presents an overview of the qualitative research questions and themes.

**Table 4:** Overview of qualitative research questions and themes

Qualitative research questions	Theme
1.What does the emotional labor of health equity work look like?	Defining health equity work
	Health equity work requires emotional labor
	Managing perceptions is part of the emotional labor of health equity work
2.What are some of the benefits and drawbacks of emotional labor in health equity work?	Health equity work is meaningful
	Collaboration impacts emotional labor costs
3.How do health equity workers cope with the emotional demands, and what more do they need?	Health equity work requires self-care
	Personal support matters
	Professional support matters

#### Defining Health Equity Work

Interviews began with the research team sharing a definition of “health equity” derived from Braveman [4p366]: “Equity means justice. Health equity is the principle or goal that motivates efforts to eliminate disparities in health between groups of people who are economically or socially worse-off than their better-off counterparts.” Interviewees frequently responded to this definition, offering their own insights and definitions. In these discussions, they offered unsolicited valuable glimpses into our first question about what health equity work involves, emphasizing a wholistic view that seeks justice in all spheres of life.

“When I think of the work that I do it's really addressing historical impact of racism on systems that have created inequities and led to us needing to do health equity work because we've had some very terrible health outcomes. And so again, my health equity work is rooted in addressing racism and its historical impact and legacy on health and life, period.”

“We are looking at the root causes of the differences in health status for populations. We are looking to both identify where there is disproportionate impact on certain groups, as well as identify the root causes and identify possible solutions…. be it transportation, housing, education, land use, planning departments the whole nine yards in order to influence the policies and practices and programs that are implemented in those spheres.”

#### Health Equity Work Requires Emotional Labor

When asked directly, interviewees uniformly believed that heath equity work involved intense emotional labor. Additionally, the interviewees felt it was important to understand and address the emotional toll health equity work takes. As one interviewee said, “When you do this work, and you feel it on that cell level… I don't know what would be adequate to cope with the emotional demands… there are pieces of it you always hold within you. Part of that emotional demand you can't put anywhere.” Multiple other interviewees had similar remarks, discussing the difficulty of coping and a tendency to suppress their emotions; a coping mechanism they found troubling.

“All of those things are emotionally hard because the weight of the injustice of all of this and the suffering that inflicts on people is just hard and there's just no getting around that. The only way I think of getting around it is if you're willing to cut yourself off emotionally and then you know that's a whole other problem.”

“We just kind of want to sweep the feelings under the rug and just say I have to keep going. You know, I have to keep going… have to do the thing.”

“You know, I thought that when I got old it would get easier, but it hasn't. It's actually gotten a little bit more difficult. I think, you know, coping... I don't know how I cope. I try to, you know, not think about it as much…”

For equity workers who identify as a member of community groups experiencing inequities, the emotional labor was even more demanding.

“As part of the group that experiences compromised outcomes, it’s impossible not to internalize some of what you feel is unfair… it’s more than a personal reaction because you see the way it’s influenced your family: your nieces, your aunts, your parents, your grandparents, your great grandparents, and so it can be [pause] tough to deal with.”

White public health equity workers also discussed struggling with the emotional burdens of their work.

“But yes, my health equity work gets very emotional for me… because you know, we still cause harm, whether we mean to or not. And so, I think part of the emotional work, if you're white, is really accepting that you will cause harm, that it doesn't make you a bad person and that you still have to be accountable for the harm.”

Finally, managers and interviewees in positions of executive leadership articulated the emotional burden they witnessed among their staff and expressed difficulty in knowing how to better support their equity workforce.

“[How do you] support people through it and not reinjure people and make progress… and still support families? It's exhausting. I mean, it's part of probably why I’m on medical leave and why we've had so many people go out on medical leave of late because it's tremendously burdensome to do this work. And to explore all this very painful stuff.”

“Our staff are tired, they’re burnt out, they’re depressed, they’re not just tired, they’re exhausted, you know? And I’m like demanding more like, ‘Okay yah, this is good but…’ And so, it’s like trying to figure out how are we creative about this? Because we don’t want to just run people into the ground kind of thing. And how can we still be inspirational and motivational for people to really get them to understand this is why we are doing this?”

#### Managing Perceptions is Part of the Emotional Labor of Health Equity Work

During interviews, participants frequently mentioned the importance and difficulty of managing perceptions. They frequently mentioned working to manage the perceptions others had about groups who experience health inequities.

“When I keep hearing people say, ‘if they would just… if those people would just…’ like trying to place blame on the individual, trying to make it seem like a woman does not want to have a healthy pregnancy and a full-term baby and her baby survive and thrive… You know, I had to at that moment, like step back and try to empathize or put myself in this person's shoes to say, like, why would he think that way?”

“I've really been kind of trying to make it my mission to, like I said, about this perspective transformation, trying to get people to understand that people are limited by the choices that they have.”

Interviewees also discussed the burden of managing perceptions others held about them.

“My [personal] work is to harness my passion, harness the anger, but to channel it appropriately so that I can demonstrate and show the sense of urgency because I want that to come through, but I don’t want to be written off as the angry black woman.”

“People want to say when they look at you, they want to already make an assessment of you and who you are currently in this work, especially with everything that's going on nationally, sometimes I am not, uh [pause] I'm not black enough, basically. I don't feel that I have the support of those that appear 100%, black, maybe they question my motives and who I am and what I represent as the health department, because I don't appear to be black enough. And that, to me, is very frustrating.”

#### Health Equity Work is Meaningful

Despite the high emotional cost, interviewees uniformly recognized a deep sense of the importance and personal significance of this work.

“There's a social justice aspect to it. There's wanting to help the communities. That I'm committed to, and that I love.”

“I hold this work in my being.”

“This is the work that I knew I was going to be doing and this is the work I knew I wanted to do. Public health is a vehicle for that for me… at the end of the day, I wanted to do something about social justice… this is a calling for me…”

“I feel like it is my responsibility to do what I do. It's a moral imperative for me. It's not just a paycheck, it's not just a job, you know, there's a bigger picture here. And I do feel a responsibility…”

#### Collaboration Impacts Emotional Labor Costs

Interviewees also discussed the emotional challenges associated with the collaborative nature of equity work, such as managing challenging personalities and difficult situations. Occasionally, these encounters required interviewees to know and enforce collaborative boundaries.

“There are some people that we just have to work with. And so, navigating those relationships… learning how to work with difficult people, and maybe if you need them to do a certain thing, it's learning how to finesse those relationships.”

“[We were] doing implicit bias training and… [someone] challenged me really hard and kind of made some false claims about me during the training. And it turned into a big thing. And it just really showed how the agency that she was working for was more willing to protect her than to actually do the work that they have signed on to do.”

“[Sometimes] I'm like, sir, or madam, you might not want to say that to me right now because I don't have the capacity to be strong, nice.”

In addition to noting the above challenges associated with collaboration, a few comments also emphasized potential benefits.

“One thing that I've been working on [is]… partnering with people, getting around people, and allowing myself to be influenced by people that have positioned themselves to make really big impacts. You know, the notion that iron sharpens iron.”

“What I take solace in is that the people around me, the community of African Americans, they understand.”

#### Health Equity Work Requires Self-Care

Interviewees were unanimous in their opinion that self-care is important. However, they expressed a wide range of confidence in their ability to engage in the practice and how best to conduct it.

“It can be very frustrating, exceptionally stressful at times, and I think a lot of us, including myself, don’t pay enough attention to self-care, if you will, in the process of doing this work.”

“I do feel responsible for myself. I don't think that I take the time to take care of myself as much as I should in this work, because I am so passionate about it. I can get so wrapped up that it's like all of my time and all of my energy, and all of my being is in the work.”

“I think to do this work… you have to make sure that you are pouring into yourself and that you take a responsibility to pour into yourself… reading a book on some sort of personal development or listening to podcasts that are about personal development and are just speaking life to whoever's listening…. So, if you truly want to help people, you're going to think about what you're pouring into yourself.”

“I spend time just being mad about stuff. Honestly, like, if I'm mad, I'm just gonna be mad about it. You know, sometimes I'm in a space where I can say something. And I feel like that's a way of coping, it’s cathartic… part of how I self-care and cope and heal is: I just have to feel it.”

“I pray a lot, that also helps me, sustains me, in terms of doing this work.”

#### Personal Support Matters

Interviewees stressed the importance of a personal support network. This included friends and family willing to help shoulder the emotional toll of health equity work.

“Well, I mean I'm lucky I get to talk through these things with my husband who's very attuned to this kind of work and provides a platform to sort of talk through that stuff. And, I have other friends that I can process with, both White friends and friends of color, to really think about how all this work plays out in our personal and professional lives.”

“I have good friends, good family, I have a lot of personal support and social support around me.”

“My personal life is important. I am blessed to have a wife of [many] years and a beautiful home together. My faith community has become important to me. So really finding balance and making sure that I am getting supported.”

As a final note concerning personal support networks, some interviewees expressed an availability of personal support but difficulty in heeding their advice. To quote one interviewee, “Everyone knows I'm not great at taking advantage of these things.” Another interviewee shared similar sentiments.

“I get it. You know. Everybody around me tells me—my kids, my wife, they tell me—slow down already. You know, you have to just slow down, take it easy. And, I think I understand why they say that, and they're right. They're right. I just need to, I just need to internalize it.”

#### Professional Support Matters

A final major theme to emerge from qualitative analysis centered on the importance of professional supports for equity workers. There was a shared sense among many interviewees that their workplace supports for equity work were inadequate. One interviewee replied emphatically, “I would say professionally, no. Absolutely not, they’re not adequate.” Another interviewee shared this sentiment but had a moment of pause before responding: “No. Uhm, but [long pause]—I’m just going to say no.” When pressed, this interviewee explained his pause was uncertainty about whether this kind of support should be a responsibility of his employer. Ultimately, he decided it should be and left his answer at “no.”

Other comments about the inadequacy of professional supports for equity workers included.

“We were supposed to have sort of a department wide effort to do more… there was a training that we had several years ago and that was supposed to have kind of been ongoing and it hasn't really happened… it's hard but there should have been a commitment… there's been so many fits and starts and it hasn't panned out.”

“They [my employers] don't get this… It's very sanitized, very white, and so I don't go there with false expectations thinking that I'm going to be able to establish camaraderie, friendship, love, and inspiration from them because they live in different realities, quite frankly.”

“They formed a task force within the organization for diversity, equity and inclusion. But they do a lot of talking, you know, so there's like, not a lot of action.”

One interviewee also wanted to emphasize that, as a person of color, he was often tasked with being the person within the organization to provide emotional support to others around equity work. This assignment created both a time-bound duty as well as additional emotional labor.

“And the thing with that, being again a black man or person of color, as you know, many times people expect you to be the voice of those within the organization and so again, you're tasked with creating this support within the organization… And so, implementing a lot of that support becomes challenging because they want you to do it all or you have all the answers. Again, back to that emotional toll…”

Despite a pervasive sentiment emphasizing the inadequacy of workplace supports, managers and frontline workers alike moderated their critiques by acknowledging the difficulties of providing support for equity workers.

“I think what is tricky is that we process so much with this stuff, but we don't get anywhere. And at a certain point, you have to sort of say, we can't process… we feel like if we don't process we’re somehow saying it's not important. And that's not the right message. But the processing isn't working, there's got to be something that we haven't hit on yet…”

“It is hard too because it comes down to time because we're so like – thinking about our office specifically – we're understaffed and there's no reduction in the amount of work that we have to do. If anything, there's more work that we have to do these days. Right? And so even if there was some sort of structure or way to, like, find time to cope, it would be hard. Because, you know, you're butting up against actual work demands as well.”

Inadequate professional support for equity work was not, however, a universal sentiment. Interviews also unearthed several bright spots, which might be considered promising practices for supporting equity workers. In their comments, it was clear that when an employer gets support right, their equity staff takes notice.

“In [our] workplace we have a really great mental wellness team that has implemented some healing circles and you know other kinds of activities and discussion forums for people to do, you know, art therapy or meditation or just connect with each other in a fun way that doesn't have anything to do with these hard conversations.”

“I think I'm in a really fortunate situation. I don't think everyone has this. But my boss is someone that I can talk to. He's a little bit older than me. And he's honestly been in the public health arena for a lot longer than I have. And he understands sort of city politics and the game, if you will, he understands that very well… Definitely helps, you know, in the health equity arena.”

“[My employer] has done a fantastic job in their health equity journey and so I'm blessed, I consider it a blessing to be able to support this organization and our work moving forward… it's exciting for me for sure.”

“I think [my workplace] is actually a very special place… I'm supported to do health equity work just by the environment that [my workplace] has created. I think that other things that we have in place help as well… like our flex time that we have in place and understanding that sometimes you just need time away.

Finally, interviewees offered suggestions for workplaces to consider in their efforts to better support equity workers.

“Speaking of just doing health equity work in general, people of color should be compensated for this work that they're doing, especially if they're doing the work on this deep level. It should be compensated, period… because folks are putting their health on the line to change things in a world and in a space where they didn't cause things to be this way.”

“I would like the workplace to just understand the double burden of doing health equity work as a Black person. And understand… I'm probably harming my health… and respecting and honoring that sacrifice that all Black people are making in doing health equity work.”

“Workplaces, especially in places that work in health equity, have a responsibility to their employees to support them. Because it is very, very hard work. Black and Brown people come into work carrying the burden and pain and trauma of current events, people dying, police brutality, and stuff like that. And then we're expected to show up, as if everything was normal, as if we're at 100% capacity as if we don't have anything else going on in our lives, like in our communities, or in our families or you know, at home. Some recognition of that and then some support in dealing with that would be really important.”

“Coming to mind for me right now is not expecting us to, to grind through it all. So, if we have a hard meeting or something challenging, or emotionally taxing today, we're expected to go right back into work tomorrow as if it was normal, as if it was fine. And so, I think not encouraging that grind culture would be really important.”

“I think what they could do is hire like 30 more people, that's what they could do. They could hire some more people, then maybe the lift won't be so heavy. But it takes like a year to get somebody on board. So, yes, that's what they can do to be more supportive, they can hire more staff.”

## Discussion

Our study sought to quantify, for the first time, the emotional labor involved in public health equity work. Additionally, we wanted to understand what equity work looks like in practice. To do this, we conducted a mixed methods study that began with a national survey and progressed to in-depth interviews with a subset of survey respondents.

Our quantitative findings demonstrated that public health equity work involves a great deal of emotional labor. The mean score on our emotional labor scale was 5.61 (range = 1–7). The high degree of emotional labor is concerning and was found to be associated with burnout. These findings echo that of prior research, frequently noting burnout among emotion workers [11, 14, 19]. Because our study did not measure the more concerning impacts of emotional labor noted in prior research (e.g., depression, fatigue, suicidal ideation, etc.), public health agencies should be cautious with their equity workforce until future studies can assess these possible impacts.

On a positive note, our study unearthed possible solutions to the association between emotional labor and job burnout. For example, personal efficacy (i.e., belief in one’s ability to do equity work well) was associated with increased job satisfaction, and burnout increased when equity workers reported not receiving adequate support for their emotional labor. Given these findings, public health agencies should institute programs aimed at assisting their equity workers with the emotional demands of their work. These agencies can take cues from existing literature in other fields [14, 30], which has suggested a proactive approach to assisting emotional laborers by implementing stress management programs, personal coping and emotional regulation skills training, and mental health services. Future research is needed to evaluate the impact of such offerings and develop a comprehensive model of workplace support for public health equity workers. Ideally, this research would identify critical support components and measure their impact on equity workers’ performance and well-being.

Findings from the qualitative strand of our research provided context. For example, interviewees offered a glimpse into why equity work involved high amounts of emotional labor by repeatedly referencing it as more than a job. We were struck by the amount of personal investment expressed by health equity workers. They often made comments about their equity work residing in the “being” or “soul” and that they were destined to do it. This finding may help explain the association we noted between the false face and burnout scales. If equity work is genuinely part of a person’s soul, but they cannot fully share that with others (i.e., false face), emotional stress and job burnout would result.

Our qualitative findings also helped to contextualize the support equity workers receive. For example, we noted how these workers recognized the importance of self-care but had difficulty practicing it. We also noted during interviews, the importance of personal connections, including family and friends, as key sources of support equity workers receive. Finally, we found that workplace support was uncommon but potentially helpful. Despite this shortcoming, promising practice examples and creative suggestions from interviewees demonstrated that improving support for equity workers is possible. Given the recent heightened emotional experience of public health work noted in the literature [2], it is more important than ever that public health agencies act on these suggestions by instituting supports for their equity workforce.

As a next step, we suggest a convening of key public health agencies and workforce experts to review findings from our study and develop key components of a comprehensive professional support system for the public health equity workforce. This support system could include localized components operated within each agency as well as networked components that link equity workers. Once developed, the support system should be pilot tested longitudinally for feasibility, acceptability, and efficacy. Once a suitable model is identified and tested, it should be brought to scale nationally to ensure our public health equity workforce is properly supported and as effective as possible in our shared mission to eliminate health inequities.

Our study has several limitations that should be noted. First, we examined the emotional labor of public health equity work in one subfield of public health—Maternal and Child Health. While the MCH workforce often shoulders a great deal of equity work, other public health subdisciplines (e.g., chronic disease, behavioral health, etc.) also address equity. Our study should be replicated with these public health sectors to ensure broad generalizability. Additionally, we used snowball and convenience sampling in our study, and while we achieved reach both nationally and across policy sectors, our sample could be biased in some way. This highlights the need for replication to confirm our findings. Finally, data collection for our study took place during the COVID-19 pandemic and national calls for racial justice. This was an emotional time for many, and our findings could be influenced by these events.

## Conclusions

The public health equity workforce faces ongoing challenges in its mission to ease, and ultimately eliminate health inequities. Our study indicates that these professionals are investing a great deal to realize results, at times even risking their personal well-being. Workplaces could do more to cultivate a work environment that supports the emotional labor demands, which would allow public health equity professionals to excel at their work.
